# Hot Melt Extruded Posaconazole-Based Amorphous Solid Dispersions—The Effect of Different Types of Polymers

**DOI:** 10.3390/pharmaceutics15030799

**Published:** 2023-02-28

**Authors:** Daniel Kramarczyk, Justyna Knapik-Kowalczuk, Mateusz Kurek, Witold Jamróz, Renata Jachowicz, Marian Paluch

**Affiliations:** 1 Institute of Physics, Faculty of Science and Technology, University of Silesia in Katowice, SMCEBI, 75 Pułku Piechoty 1a, 41-500 Chorzów, Poland; 2 Department of Pharmaceutical Technology and Biopharmaceutics, Faculty of Pharmacy, Jagiellonian University, Medyczna 9, 30-688 Kraków, Poland

**Keywords:** posaconazole, amorphous, amorphous solid dispersion, hot melt extrusion, physical stability, semi-crystalline polymers, amorphous polymers

## Abstract

Four model polymers, representing (i) amorphous homopolymers (Kollidon K30, K30), (ii) amorphous heteropolymers (Kollidon VA64, KVA), (iii) semi-crystalline homopolymers (Parteck MXP, PXP), and (iv) semi-crystalline heteropolymers (Kollicoat IR, KIR), were examined for their effectiveness in creating posaconazole-based amorphous solid dispersions (ASDs). Posaconazole (POS) is a triazole antifungal drug that has activity against Candida and Aspergillus species, belonging to class II of the biopharmaceutics classification system (BCS). This means that this active pharmaceutical ingredient (API) is characterized by solubility-limited bioavailability. Thus, one of the aims of its formulation as an ASD was to improve its aqueous solubility. Investigations were performed into how polymers affected the following characteristics: melting point depression of the API, miscibility and homogeneity with POS, improvement of the amorphous API’s physical stability, melt viscosity (and associated with it, drug loading), extrudability, API content in the extrudate, long term physical stability of the amorphous POS in the binary drug–polymer system (in the form of the extrudate), solubility, and dissolution rate of hot melt extrusion (HME) systems. The obtained results led us to conclude that the physical stability of the POS-based system increases with the increasing amorphousness of the employed excipient. Copolymers, compared to homopolymers, display greater homogeneity of the investigated composition. However, the enhancement in aqueous solubility was significantly higher after utilizing the homopolymeric, compared to the copolymeric, excipients. Considering all of the investigated parameters, the most effective additive in the formation of a POS-based ASD is an amorphous homopolymer—K30.

## 1. Introduction

Improving the aqueous solubility and dissolution rate of poorly water-soluble active pharmaceutical ingredients (APIs), and new chemical entities (NCEs), is currently one of the most significant difficulties facing modern pharmacy [1,2,3]. This need is related to the estimation that almost 90% of very promising NCEs, and more than 50% of currently commercialized APIs, have solubility-limited bioavailability, which makes them fall under the II or IV classes of the Biopharmaceutical Classification System (BCS) [4,5,6,7]. One of the methods that might overcome this problem, and improve the aqueous solubility of the BCS class II materials, is their amorphization [8,9,10,11].

Amorphous materials, due to a lack of long-range molecular order and, associated with that, higher internal energy, are characterized by higher apparent solubilities, faster dissolution rates, and potentially better bioavailability, when compared to their crystalline counterparts [12,13,14,15]. However, these benefits come at a cost—these systems are thermodynamically unstable and require stabilization [16,17,18]. Currently, the most common strategy for preserving the recrystallization of amorphous APIs, is to disperse them into a polymeric matrix molecularly, i.e., prepare an amorphous solid dispersion (ASD) [19,20,21]. It is generally recognized that the role of the polymer in an ASD is to reduce molecular mobility, inhibit the nucleation and crystal growth of amorphous active material, and facilitate its release from the polymeric matrix [22,23,24,25].

There are quite a large number of polymers commonly used in drug formulation that can reduce the tendency of amorphous drugs toward recrystallization, e.g., poly(vinylpyrrolidone) (PVP), poly(vinylpyrrolidone-vinyl acetate) (PVPVA), poly(styrene sulfonic acid) (PSSA), (hydroxypropyl)methyl cellulose (HPMC), hypromellose acetate succinate (HPMCAS), soluplus (SOP), poly(vinyl alcohol) (PVA), and polyethylene glycol (PEG) [26,27,28,29,30,31]. However, the choice of an appropriate stabilizer is very difficult. There are a few, sometimes opposite aspects, which one needs to take into account. For example, the chosen polymer should be appropriately soluble in water. It has been demonstrated many times that poorly water-soluble polymers may limit the amount of drug released, leading to inadequate supersaturation levels [26]. On the other hand, it has also been proven that selecting a polymer with aqueous solubility that is too high, may also not be a good choice. For example, water-soluble excipients might reveal less tendency to interact with hydrophobic drug substances and, therefore, might not be able to protect their amorphous form from fast recrystallization [32]. Another challenge worth noting, is assessing the adequate polymer content in an ASD [33,34]. On the one hand, the smaller the amount of polymer, the greater the risk of API recrystallization. Thus, from the stabilization point of view, the higher the polymer amount, the better, but, on the other hand, too high a polymer content means increased dosage size, which can make it difficult for the patient to swallow the oral dosage form. Taking into account all of these criteria, it is not surprising that designing an ASD possessing an appropriate balance of active substance and polymer is a complex problem. The case is even more complicated when adding parameters important from the production point of view to the criteria [35,36,37].

On a large scale, ASDs are usually produced using either spray drying (SD) or hot melt extrusion (HME) [38,39,40,41]. However, due to its various advantages, such as there being no organic solvents in the processing, the small footprint of the equipment, the ease of increasing the batch size, and consequent scalability from pilot to industrial setting, HME has become a more and more preferred method in the development of ASDs [42,43,44,45]. In this process, the most critical parameters are, production temperature and material viscosity. Overheating may result in the thermal decomposition of the active substance [46]. At the same time, the employment of too low a temperature might not be sufficient to obtain a fully amorphous system, i.e., melt the drug [47,48]. Furthermore, in this particular—not too high and not too low—temperature region, the drug–polymer composition needs to be characterized by a viscosity in the range of 800–10,000 Pa·s [19,49]. This value corresponds to the generally accepted “rule-of-thumb viscosity range” for small-scale extrusion.

The goal of this work was to check the efficiency of four well-known polymers: Kollidon 30 (K30) [50], Kollidon VA64 (KVA) [51,52,53], Parteck MXP (PXP) [53,54,55], and Kollicoat IR (KIR) [23,56] in the formation of an ASD containing posaconazole (POS) [57]. POS is a representative of triazoles, i.e., substances used as first-line treatments against fungal infections. The advantage of this API, compared to other azoles (itraconazole, fluconazole, voriconazole), is that it has a wider spectrum of activity, and a more favorable safety profile. It is commercially available in the form of Noxafil, in the form of an oral suspension or tablets. Due to its low solubility, i.e., <1 μg/mL, POS is classified as BCS class II. Therefore, there is a need to improve its solubility, which could be achieved by the conversion of POS to an amorphous form. This solution requires us to use excipients, to maintain the physical stability of POS and the appropriate viscosity for the HME process, which is ensured by additives in the form of polymers, creating ASDs. Noxafil tablets also contain an amorphous solid dispersion of posaconazole, however, due to limited drug loading, patients have to take up to three tablets to take a standard dose (300 mg) of posaconazole. That is why searching for a stable ASD, with a higher drug loading, is still necessary.

**Figure 1:**
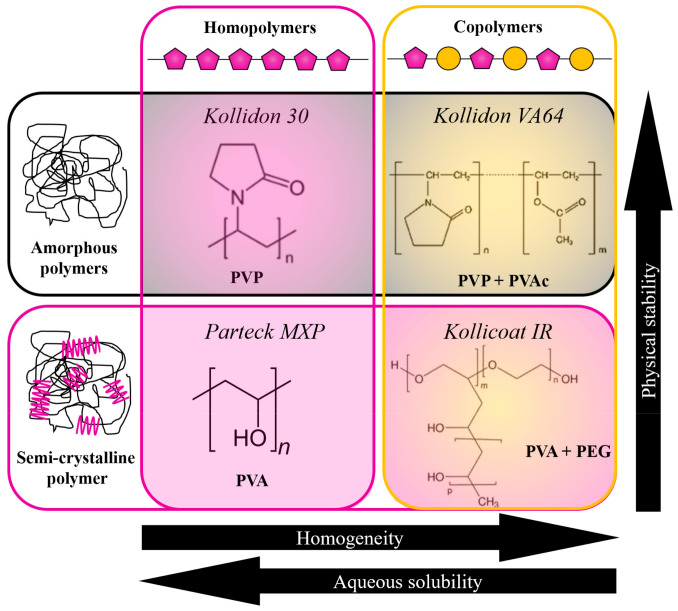
Schematic illustration of the types of polymers investigated in this work for POS-based ASD.

The most important aspects which were checked in this paper were: (i) the amorphous POS stabilization efficiency, (ii) the possibility of manufacturing ASDs by HME, (iii) drug loading, (iv) improvement of the API’s aqueous solubility, and (v) modifications in its dissolution rate. It is worth noting that selecting these polymers allows the assessment of the usefulness of different types of polymers in forming ASDs. As summarized in Figure 1, depending on grouping, the chosen polymers might represent amorphous or semi-crystalline polymers, and homo- or copolymers. Taking into account the structural similarities of Kollidon 30 and Kollidon VA64, as well as Parteck MXP and Kollicoat IR, we will also discuss what impact PVAc- or PEG-mers have on the investigated properties of the POS-based ASDs. Furthermore, due to the large variation in the glass transition temperature (T_g_) of the chosen polymers, its impact will also be discussed. The thermal and viscoelastic properties of POS-based compositions, possessing various polymer content, were investigated to achieve some of the aforementioned goals. Next, the selected concentrations of each drug–polymer composition were extruded. Finally, the thermal properties, long-term physical stabilities, water solubilities, and dissolution rates of the obtained extrudates were investigated. The ultimate aim of this comparative study was to provide general guidance for the screening of polymers suitable for HME.

## 2. Materials and Methods

### 2.1. Materials

Posaconazole (POS, > 99.5% purity, 4-[4-[4-[4-[[(3R,5R)-5-(2,4-difluorophenyl)tetrahydro- 5-(1H-1,2,4-triazol-1-ylmethyl)-3-furanyl]methoxy]phenyl]-1-piperazinyl]phenyl]-2-[(1S,2S)-1-ethyl- 2-hydroxypropyl]-2,4-dihydro-3H-1,2,4-triazol-3-one, Wuhan ChemNorm Biotech Co., Ltd., Wuhan, China) was used as a model drug. Two amorphous polymers, i.e., Kollidon K30 (K30, poly(vinylpyrrolidone), and Kollidon VA64 (KVA, poly(vinylpyrrolidone-vinyl acetate)), both from BASF, Ludwigshafen am Rhein, Germany, and two semi-crystalline polymers, Kollicoat IR (KIR, Macrogol poly(vinyl alcohol)Grafted Copolymer, BASF, Ludwigshafen am Rhein, Germany), and Parteck MXP (PXP, poly(vinyl alcohol), Merck KGaA, Darmstadt, Germany) were used as polymeric matrices for ASD. The physical mixtures, containing crystalline POS and one of the listed polymers in concentrations from 10 to 70 wt.% of the polymer, were prepared by co-mixing the appropriate amount of the materials inside an agate mortar. The binary systems, containing an amorphous form of POS, were obtained after the physical mixture’s melt quench. The water used in all tests was produced by an Elix 15UV Essential reversed osmosis system (Merck KGaA, Darmstadt, Germany). All other reagents were of the analytical grade.

### 2.2. Thermogravimetric Analysis (TGA)

The thermal stabilities of both neat POS and the employed polymers, as well as their physical mixtures, were investigated by a Mettler TG 50 thermogravimetric analyzer (Mettler-Toledo, Greifensee, Switzerland) linked to a Mettler MT5 balance (Mettler-Toledo, Greifensee, Switzerland). The powder, in open aluminum pans, was placed in a furnace under nitrogen purge (60 mL/min), and heated at 10 K/min. Degradation of the sample was determined in the temperature range from 303 to 723 K, by the weight loss percentage. The data obtained from TGA are attached in the Supplementary Materials as Figures S1 and S2.

### 2.3. Differential Scanning Calorimetry (DSC)

The thermal properties of all investigated POS-based binary systems were measured using the Mettler Tolledo STAR^e^ system (Columbus, OH, USA), equipped with an HSS8 ceramic sensor, 120 thermocouples, and the liquid nitrogen cooling accessory. The measuring device was calibrated for temperature and enthalpy using zinc and indium standards. The samples were placed in an aluminum crucible (40 μL). Both the initial, i.e., neat crystalline POS, as well the binary drug–polymer systems, containing crystalline API, were heated up from 298 to 453 K (in the case of the systems with K30 or KVA) or to 488 K (in the case of the systems with PXP), or to 503 K (in the case of the systems with KIR), with a rate of 10 K/min. Systems containing amorphous POS were heated up from 298 to 468 K (in the case of systems with K30 and KVA) or to 488 K (in the case of systems with PXP), as well as from 183 to 503 K (in the case of systems with KIR), with the same rate. The thermal properties of the as-obtained, and long-term stored, extrudates were investigated in a few ways. The samples were measured from 278 to 488 K (when containing amorphous polymers), or to 503 K (when containing semi-crystalline polymers), (i)without an annealing procedure prior to the heating step, with a rate of 10 K/min, through the standard DSC technique, (ii) with a 30 min annealing procedure at T = 373 K prior the heating step, with a rate of 10 K/min, through the standard DSC technique, and (iii) with a 30 min annealing procedure at 353 K (in the case of systems containing KVA), or at 323 K (in the case of systems containing PXP and KIR), prior to the heating step, with a rate of 10 K/min, through the standard DSC technique. It needs to be pointed out that, just before each experiment, the extrudates (stored at controlled conditions: T = 298 K and RH = 25%, inside a glove box (PLAS Laboratories Inc. 890-THC-DT/EXP/SP)) were minced in the agate mortar. The beginnings of the exothermal and endothermal peaks were identified as the crystallization and melting temperatures, respectively. At the same time, the glass transition temperature (T_g_) was determined as the midpoint of the heat capacity increment. Each measurement was performed in triplicate (*n* = 3). Average values and standard deviations (SDs) were calculated.

### 2.4. Rheology

The viscoelastic properties of binary drug–polymer ASDs, containing 50, 60, or 70% KVA polymer, or 40, 50, or 60% K30 or KIR or PXP polymer, were measured utilizing an ARES G2 Rheometer, using an aluminum parallel plate geometry (diameter = 25 mm); the gap was 1 mm. The temperature sweep measurements were conducted at a constant frequency of 1.5915 Hz, and the strain was within the linear viscoelastic region. The samples, in the form of amorphous solid dispersions, were cooled down (i) from 443 to 393 K for POS + KVA systems, (ii) from 453 to 393 K for POS + K30 and POS + KIR systems, and (iii) from 473 to 393 K for POS + PXP systems. A frequency range from 0.016 to 7.958 Hz was utilized.

### 2.5. Hot-Melt Extrusion (HME)

HME processes were performed using a 40D, 12 mm corotating twin-screw extruder (RES-2P/12A Explorer, Zamak Mercator^®^, Skawina, Poland), equipped with an air-cooled conveying belt (Zamak Mercator^®^, Skawina, Poland). The mixtures of POS and matrix-forming polymers, i.e., K30, KIR, and PXP, in a 50:50 mass ratio, and a mixture with KVA in a 30:70 mass ratio, were extruded through a 1.75 mm die at 50 rpm screw speed. We used standard parallel, corotating, fully intermeshing screws, with three kneading zones, to mix the API and polymer properly. The detailed configuration of the screws was presented in our previous study [58]. The entire barrel temperature was maintained at 463 K for the POS + PXP mixture extrusion. All other extrusions were performed at 443 K. The batches extruded were 50 g. As the powder blends were characterized by poor flow, they were manually fed. The feeding rate, calculated based on batch size and process duration, was in the range of approximately 3–5 g/min. During the extrusion, the individual heating zones’ temperatures, die pressures, torques, and engine loads were monitored, to evaluate the process.

### 2.6. High-Performance Liquid Chromatography (HPLC)

An Agilent 1200 Infinity (Waldbronn, Germany), equipped with a diode array detector and a Kinetex C18 reverse phase LC column Kinetex C18 (100 × 4.6 mm; particle size 2.6 μm; pore size 10 nm, Phenomenex), were used for analyses. The mobile phase consisted of acetonitrile and a 15 mM potassium dihydrogen phosphate solution (pH = 4.7), ran in an isocratic elution in 55:45 (*v*/*v*) ratio, at a flow rate of 1.5 mL per minute. The column temperature was maintained at 313 K. The injection volume was equal to 20 μL. The total run time was 3 min. The retention time of POS was 1.8 min. POS detection was carried out at λ = 262 nm. Linearity was confirmed within the concentration range of 7.8–54.4 μg/mL (R^2^ = 0.9997). Each measurement was performed in triplicate (*n* = 3). Average values and standard deviations (SDs) were calculated.

### 2.7. Equilibrium Solubility

An excess of crystalline POS, amorphous POS (prepared by quench cooling), milled extrudates, and physical mixtures of POS with polymers (equivalent to 5 mg of POS) were dispersed in 2 mL of distilled water (pH = 5.46). The suspensions were shaken at 400 rpm, at ambient temperature (approx. 295 K), for 48 h, using the Unimax 1010 orbital shaker (Heidolph Instruments GmbH & Co. KG, Schwabach, Germany), until equilibrium was reached. The rotational speed was selected to achieve proper mixing of the suspension and to avoid sedimentation, which can limit the dissolution. The samples were centrifuged at 3600× *g* rpm for 10 min in the MPW 221 centrifuge (MPW MED Instruments, Warsaw, Poland), and filtered through a nylon 0.22 µm KX syringe filter (Kinesis). The samples were analyzed in triplicate using the HPLC method. The reported data represent the averages of three series of measurements with standard deviations (SD).

### 2.8. Dissolution Studies

The dissolution of POS from extruded polymer matrices was performed in the DFZ 60 flow-through apparatus (USP Apparatus 4), with an HKP 60 pump (ERWEKA GmbH, Langen, Germany). The extruded material was milled with a Tube Mill 100 (IKA-Werke GmbH & Co. KG, Staufen, Germany). The 100–200 µm particle fraction was used in dissolution studies, to avoid the impact of particle size on dissolution profiles. A single 5 mm glass bead was placed at the bottom of the large flow-through cell for tablets and capsules, and the cell cone was then filled with 2 mm glass beads to achieve constant and laminar dissolution medium flow. The milled extrudates were placed on top of the glass beads and covered with another batch (2 g) of 2 mm glass beads, to prevent the powder from floating and sticking together. A constant 16 mL/min flow of water (pH = 5.46), maintained at 310 K, was used in an open-loop configuration. The samples were collected at predetermined timepoints, and the concentration of POS was assayed by HPLC. All dissolution studies were carried out in triplicate (*n* = 3). Average values and standard deviations (SDs) were calculated.

### 2.9. Posaconazole Content

Accurately weighed samples of milled extrudates were introduced to 25 mL flasks and shaken, with a mixture of water and methanol in a 20:80 ratio, for 24 h, using the Unimax 1010 orbital shaker (Heidolph Instruments GmbH & Co. KG, Schwabach, Germany) at 300 rpm. Afterward, the samples were filtered through a nylon 0.22 µm KX syringe filter (Kinesis), and the amount of POS was determined by HPLC.

## 3. Results and Discussion

### 3.1. Thermal Properties of POS-Based Binary Systems

**Figure 2:**
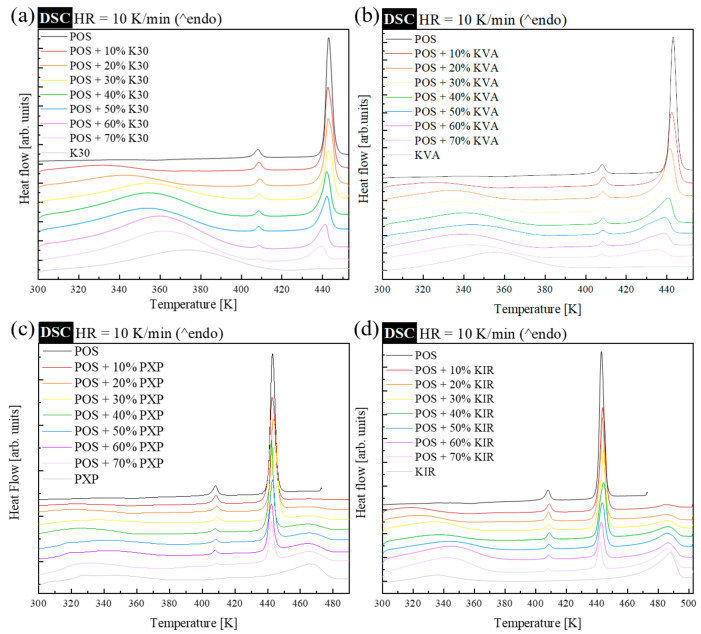
DSC thermograms of physical mixtures containing crystalline POS and, from 10–70 wt.%, of (**a**) Kollidon 30 (K30), (**b**) Kollidon VA64 (KVA), (**c**) Parteck MXP (PXP), and (**d**) Kollicoat IR (KIR). In each panel, the uppermost thermogram represents a DSC trace of neat crystalline (as-received) POS, while the lowest thermogram originates from neat (as-received) polymer.

In the first step of our studies, the impact of the chosen polymers (at concentrations of 10, 20, 30, 40, 50, 60, and 70%) on the melting temperature of POS was investigated. For that purpose, non-isothermal DSC experiments of the physical mixtures were performed. During these experiments, the samples were heated at a rate of 10 K/min. All compositions were investigated at 298 K, while the final temperature depended on the polymer employed. For compositions containing K30 or KVA, the sample was heated up to 453 K. In the case of the systems with PXP or KIR, the final temperature was equal to 488 or 503 K, respectively. Each sample was investigated in triplicate. The representative DSC traces are summarized in Figure 2. To make the analysis clearer, in each panel of Figure 2, the DSC thermograms of as-received neat crystalline POS, and neat polymers, have been added.

As can be seen, the DSC thermogram of neat crystalline POS is characterized by two endothermal processes, which correspond to the melting of the II and I polymorphic forms of this active substance. On the DSC traces of POS + K30 and POS + KVA, one can notice as many as three, while on the DSC traces of POS + PXP and POS + KIR, one can notice four endothermal events. Looking from low to high temperatures, the first endothermic process, in all investigated systems, reflects water evaporation, the second (at ca. 408 K) and the third (at ca. 440 K) events are associated with the melting of the II and I polymorphic forms of POS, respectively. The fourth endothermic process, which is visible only for systems with PXP and KIR, is associated with the melting of the crystalline fraction of the employed semi-crystalline polymers.

It is worth noting that, in the case of the POS-based systems containing amorphous polymers (i.e., K30 or KVA), the melting temperature decreases with increasing polymer content. While in compositions containing the semi-crystalline polymers (i.e., PXP or KIR), the lack of a significant depression of the drug melting point is noted. Since depression of the melting point only manifests in miscible drug–polymer combinations, it is reasonable to assume that POS is immiscible with the semi-crystalline polymers used. It is well known that, if the API and polymer are immiscible, melting point depression will not occur, because the presence of the excipient does not alter the chemical potential of the active substance. The possible lack of miscibility is not the only drawback of semi-crystalline polymers employed in POS-based ASDs. Another disadvantage of these polymers is that, due to a higher melting point than that of neat POS, to disperse the API in the polymer matrix utilizing HME molecularly, such systems require heating of the API to above its melting point. Usually, HME polymers must lower the drug’s melting point, to reduce the possibility of thermal decomposition. Thus, when assessing the effectiveness of the employed polymers in lowering the POS melting temperature (which is equivalent to the suitability of the polymer in HME), they can be ranked as follows: Kollidon VA64 > Kollidon 30 > Parteck MXP > Kollicoat IR.

**Figure 3:**
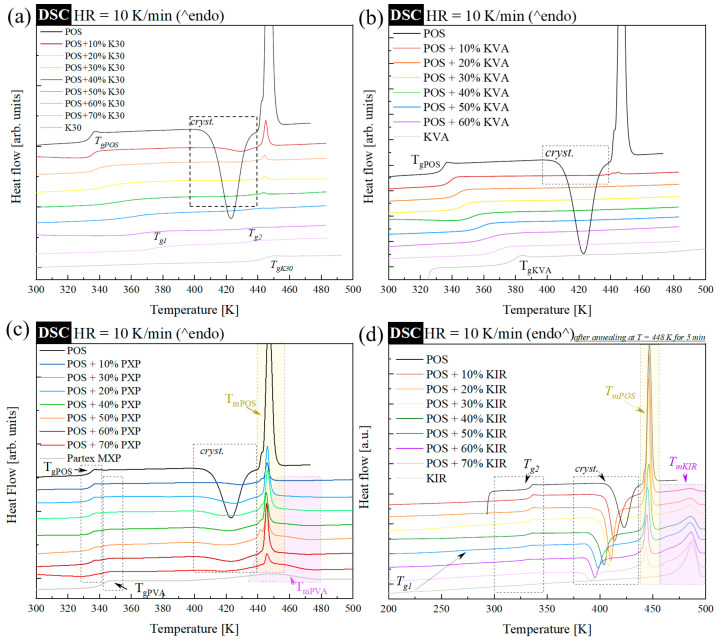
DSC thermograms of melt quenched binary systems containing amorphous POS, and from 10–70 wt.% of (**a**) Kollidon 30 (K30), (**b**) Kollidon VA64 (KVA), (**c**) Parteck MXP (PXP), and (**d**) Kollicoat IR (KIR). In each panel, the uppermost thermogram represents the DSC trace of amorphous POS, while the lowest thermogram originates from neat (dry) polymer.

Evidently, the reduction in the API melting point by the polymer is not the only important parameter that should be considered when designing ASDs. Therefore, to (i) estimate the tendency of the employed polymers to form miscible systems with POS, and (ii) assess how the excipients improve the physical stability of the API, the systems were quench-cooled in situ, using DSC, and the glasses obtained in that way were heated up. The quench cooling procedure was performed at a rate of 20 K/min, from a starting temperature of: (i) T = 448 K for systems containing POS and K30 or KVA, (ii) T = 468 K, with 2 min annealing, for POS and PXP systems, and (iii) T = 503 K, as well as T = 448 K with 5 min annealing (reduced temperature due to thermal decomposition observed at T = 503 K), for POS and KIR systems. Besides the systems containing KIR, where the cooling procedure was taken down to T = 183 K, the drug–polymer systems were cooled down and reheated from T = 298 K. Similar to the previous studies, all samples were investigated in triplicate. The representative DSC traces of quench-cooled binary compositions are presented in Figure 3. Each panel includes DSC thermograms of neat quench-cooled POS and polymer, to facilitate the analysis.

As can be seen, the DSC trace of neat amorphous POS reveals a glass transition event at T_g_ = 334 K. During further heating, with a rate of 10 K/min, one might note the exothermal process associated with cold crystallization, followed by an endothermal event reflecting melting of the recrystallized part of the API. Based on this information, POS might be classified as a class II material, according to the approach proposed by J. A. Baird [59]. These materials do not display crystallization during cooling (20 K/min), but their recrystallization is observed following the reheating step (10 K/min). Consequently, such systems are rated as good glass formers, with a high tendency toward recrystallization. The observed low physical stability of the amorphous POS was one of the reasons for selecting this compound for our studies. Fast recrystallization guarantees the possibility of a quicker assessment of the effectiveness of polymers in improving the API’s physical stability.

The thermograms of the neat, dry polymers K30, KVA, and PXP, shown as gray lines in Figure 3a–c, respectively, are characterized by one glass transition event, located at T_g_ = 443, 376, and 341 K, respectively. In the case of KIR, the situation is more complex, since two T_g_s were registered—the first at T_g1_ = 217 K, and the second at T_g2_ = 319 K (see thermogram presented as a gray line in Figure 3d). The presence of T_g1_ in KIR was the main reason why all the samples containing this polymer were investigated from T = 183 K. Except for the glass transition events on the thermograms of PXP and KIR, the melting endotherm (located at T_m_ = 441 K for PXP, and T_m_ = 468 K for KIR) can be noted, proving the semi-crystalline nature of these polymers. It is worth noting that all of the above-determined T_g_ and T_m_ values agree well with the literature data.

Comparing the DSC thermograms of the POS + K30 systems, one can divide these compositions into two groups—one containing the polymer in concentrations up to 40%, and the other containing the polymer in concentrations above 40%. Compositions with less than or equal to 40% content of the investigated amorphous homopolymer, display three thermal events. The first, located at the lowest temperature, has step-like behavior, and is associated with the glass transition event. The second is the exothermal event, reflecting the POS recrystallization. While the third is the endothermal event, connected with the melting of the recrystallized fraction of POS. On the thermograms of compositions containing 50% or more of K30, a lack of a recrystallization process (and associated with this, a melting process) can be clearly noted. At the same time, these thermograms reveal the second glass transition event (T_g2_), which, in systems with lower polymer concentrations, might be possibly covered by the crystallization exotherm (see Figure S3, presenting the zoomed area of Figure 3a). This result suggests that the investigated POS + K30 systems are not entirely homogeneous. If components are homogeneous, the DSC curve of an amorphous composition should reveal a single glass transition event. Since, however, with increasing the polymer content, the T_g_ located at the lower temperature shifts toward higher temperatures, one might assume that partial homogenization is reached, and two—API-reach and polymer-reach—regions are formed. This might mean that POS and K30 are miscible, but do not form fully homogeneous systems after quenching their physical mixtures.

Lack of phase separation was noted in the systems containing POS and KVA (amorphous copolymer). As seen in Figure 3b, the DSC thermograms of all investigated concentrations containing POS and KVA display only one glass transition event, which shifts toward higher temperatures with increasing polymer content. POS in the drug–polymer compositions containing the investigated amorphous copolymer also shows a lower tendency toward recrystallization, compared to compositions containing the investigated amorphous homopolymer (K30). The crystallization exotherms were noted on DSC thermograms only for POS + 10% KVA (see Figure S4, presenting the zoomed area of Figure 3b).

Figure 3c, shows the thermograms of the quench-cooled POS and PXP systems. Herein, one can note the lack of modification in the glass transition event of POS in the presence of the semi-crystalline homopolymer (see Figure S5, presenting the zoomed area of Figure 3c). The thermograms at all investigated concentrations reveal two overlapping glass transitions, originating from the T_g_s of API and polymer. The glass transitions’ heat capacities (ΔC_p_) vary depending on the drug–polymer concentration. It is also worth noting that, at all investigated concentrations of the system containing PXP, one can notice that POS recrystallizes. This is manifested by the presence of the exothermal process in the vicinity of 400–440 K on the DSC thermograms. This process is followed by two overlapping endothermal events, reflecting the melting of POS and the crystalline fraction of the polymer. Because of the lack of influence of both materials on each other, described above, is clear (lack of significant shifts in T_g_, T_c_, and T_m_), one can expect that POS and PXP are not miscible.

The last POS-based composition investigated in this work contains KIR—the semi-crystalline copolymer. As seen in Figure 3d, this polymer plasticizes the POS, i.e., shifts its glass transition temperature toward lower temperatures with increasing polymer content. The observed effect does not prove that the composition of the formulation containing POS and KIR is entirely homogeneous. Looking more precisely, one can notice an additional glass transition event (barely seen on the presented thermograms) located at lower temperatures, that originates from the KIR-reached fraction of the composition. However, it needs to be stressed that the difficulty in achieving full homogeneity in systems containing KIR, can be associated with the fact that the neat KIR polymer also exhibits two glass transitions (see Figure S6, presenting the zoomed area of Figure 3d). Thus, such behavior is commonly seen in formulations containing this polymer. Apart from the glass transitions, all investigated concentrations of POS + KIR displayed on the DSC thermogram, one clearly visible exothermal process, originating from the recrystallization of POS, followed by two melting endotherms, separated from each other. The first melting originates from the POS, while the second is associated with the KIR’s liquefying. It is clear that, with increasing polymer content, the temperature of POS’s crystallization onset decreases. This result suggests that the POS composition with KIR is the least physically stable of all of the investigated ASD. Therefore, when assessing the effectiveness of the employed polymers’ suitability for forming HME POS-based ASDs, one should rank them as follows: KVA (miscible and homogeneous, the highest melting point depression, the highest physical stability) > K30 (miscible but nonhomogeneous, visible melting point depression, visible POS stabilization) > PXP (immiscible, lack of melting point depression, lack of stabilization) > KIR (miscible but nonhomogeneous, lack of melting point depression, lack of stabilization).

**Figure 4:**
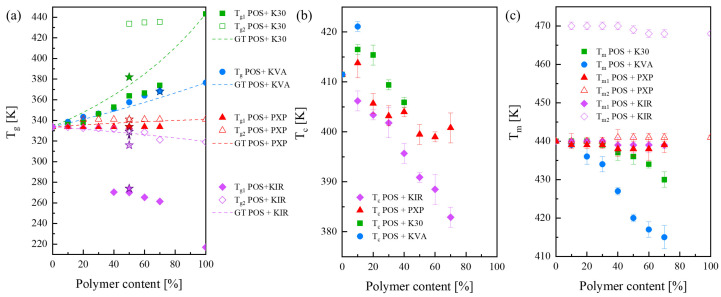
Concentration dependences of (**a**) glass transition temperature, (**b**) crystallization onset, (**c**) melting point of the investigated POS + K30 (green squares), POS + KVA (blue circles), POS + PXP (red triangles), and POS + KIR (purple diamonds). The star symbols represent the *T_g_* values of systems obtained after the HME process.

To better visualize how the employed polymers modify the glass transition, the onset of crystallization, and the melting temperature of POS, these data are summarized in Figure 4a–c, respectively. In Figure 4a, the theoretically determined concentration dependences of T_g_ for each system are also presented. The dependencies have been calculated based on the Gordon and Taylor approach [60,61]:

(1) $$T_{g}=\frac{W_{1}T_{g1}+KW_{2}T_{g2}}{W_{1}+KW_{2}}.$$

where *w*_1_ and *w*_2_ are the weight fractions of each component, and *T_g_*_1_ and *T_g_*_2_ correspond to the glass transition temperatures of each component. *T_g_* is the glass transition temperature of the mixture, while *K* is a measure of the interaction between the components, and can be defined as follows:

(2) $$K\approx \frac{∆C_{p2}}{∆C_{p1}}.$$

Δ*C_p_*_1_ and Δ*C_p_*_2_ in Equation (2) denote the change in heat capacity at the glass transition temperature of each component (in the case of systems containing KIR, only the data from *T_g_*_2_ were taken).

From all of the data that has been collected so far, it appears that KVA is the best polymer to create ASD with POS. This amorphous copolymer provides the highest physical stability of the amorphous form, easily forms a homogeneous system with the API, and gives the possibility of employing the lowest production temperature, compared to the other polymers. K30 ranks second, since it can reduce the melting point of POS and stabilize the API when a higher polymer content is employed. Of course, these improvements are not as impressive as in the case of KVA. PXP ranks third, because it does not facilitate amorphous POS’s recrystallization as well as KIR. However, both these semi-crystalline polymers display a lack of (i) homogenization with POS, (ii) significant improvement of the API’s physical stability, and (iii) the depression of POS’s melting point. Based on that, one can conclude that amorphous polymers are undoubtedly better stabilizers of amorphous APIs than semi-crystalline polymers. In the following research stage, the rheological characteristics of the chosen binary systems were examined, to ascertain whether or not KVA would still be the best excipient, offering the appropriate viscosity for producing POS-based ASDs by HME.

### 3.2. Effect of Temperature on Melt Viscosity of POS-Based Systems

**Figure 5:**
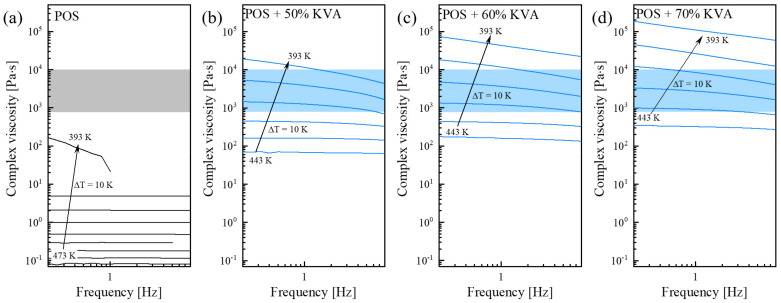
Complex viscosity as a function of frequency, measured at various temperature conditions, for (**a**) neat POS, (**b**) POS + 50% KVA, (**c**) POS + 60% KVA, and (**d**) POS + 70% KVA. The shaded area indicates the generally accepted “rule–of–thumb viscosity range” for small–scale extrusion.

In this section, the rheological properties of neat POS, and its ASDs containing 50, 60, and 70% of KVA, and 40, 50, and 60% of K30, PXP, or KIR, were investigated, for the selection of one suitable for HME processing temperatures. Temperature sweep measurements were performed at fixed frequencies (0.016–7.958 Hz) for that purpose. These experiments were performed at temperatures (a) from 473 to 393 K (ΔT = 10 K) for systems with neat POS, (b) from 443 to 393 K (ΔT = 10 K) for systems with KVA, (c) from 453 to 393 K (ΔT = 10 K) for systems with K30, (d) from 473 to 373 K (ΔT = 10 K) for systems with PXP, and (e) from 453 to 403 K (ΔT = 10 K) for systems with KIR. The representative data (for neat POS and its systems with KVA) are presented in Figure 5. The shaded areas represent the generally accepted “rule-of-thumb viscosity range” for small-scale extrusion (that is 1800−10,000 Pa·s). The viscosity of neat POS at the temperature of its melting region is far below that appropriate for the HME viscosity range. This means that, to obtain a system suitable for HME containing POS, some additive improving its viscosity would be needed. For that, and also other purposes such as physical stability and water solubility improvement, the employed polymers were utilized.

**Figure 6:**
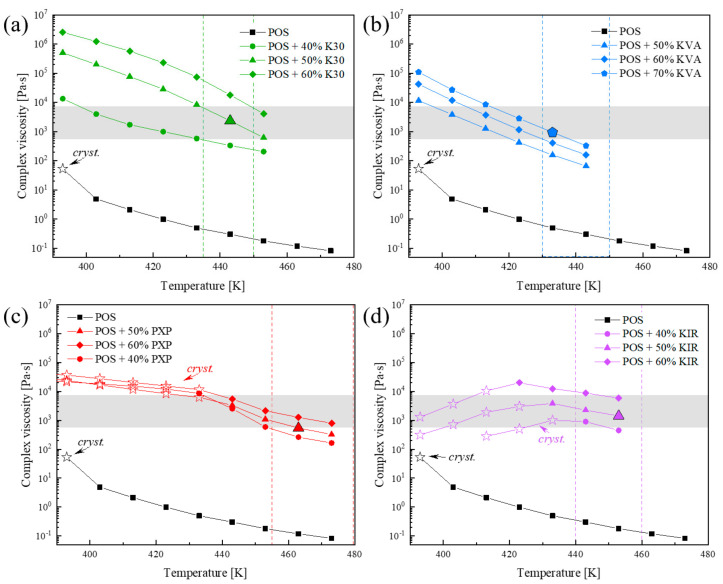
Oscillation temperature sweep of binary drug–polymer mixtures of (**a**) POS + K30, (**b**) POS + K30, (**c**) POS + PXP, and (**d**) POS + KIR from 443 K (for POS + KVA), 453 K (for POS + K30 and POS + KIR), and 473 K for (POS + PXP), to 393 K, at an angular frequency of 1 Hz.

Figure 6 compares the effect of temperature on melt viscosity for all investigated POS-based systems, using the viscosity values determined at f = 1 Hz. As can be seen, samples become more viscous when the temperature decreases, or polymer content increases. In the case of neat POS, and systems containing PXP or KIR polymer, the POS’s recrystallization was observed during sample cooling, proving these materials’ low physical stabilities. The viscosity values determined from partially crystalline systems are marked as star symbols. Figure 6 also shows the area where each tested system melts (see areas between dashed lines, which were obtained based on calorimetric data). By correlating these values with viscosity values suitable for HME (i.e., ‘shaded area’), it is easy to determine which of the tested concentrations of given API + polymer systems are the most suitable for the extrusion process. Comparing the effect of investigated amorphous polymers on the melt viscosity of POS, it is seen that about a 20% lower amount of K30, which is more viscous than KVA, is needed to obtain a binary drug–polymer system with the appropriate characteristics of extrusion melt viscosity. While, to make POS’s melt viscosity suitable for HME in the binary systems containing any of the employed semi-crystalline polymers (KIR or PXP), nearly the same amount of the additive is required. Summarizing, employing as much as 70% KVA or 50% K30, PXP, or KIR is necessary to make POS’s viscosity in its melting temperature area appropriate for HME.

Due to the significantly lower drug loading in systems with KVA, compared to the other compositions, these results might modify the rankings presented in the previous section, of the polymers’ effectiveness in forming the best POS-based ASD. A high drug loading, with simultaneous high physical stability, makes POS + K30 a potentially better system than POS + KVA. The compositions employing the semi-crystalline polymers—PXP and KIR—also required less polymer (i.e., 50%) than KVA to ensure an appropriate viscosity of POS. These polymers, however, do not improve the physical stability of amorphous forms of POS. A drawback of the employment of PXP, KIR, and even K30 in formulations with POS worth noting is, the lack of complete homogeneity (described in the last section) or miscibility, as in the case of POS + PXP. However, it is well known that the sample preparation method might impact its homogeneity. Thus, we decided to prepare the extrudates (i.e., melt-mixed compositions) of the investigated API + polymer systems in the concentrations that had provided the viscosity values most appropriate for HME, i.e., POS + 50% K30, POS + 70% KVA, POS + 50% PXP, and POS + 50% KIR. The main aim of these efforts was to check whether it was possible to obtain better homogeneity of the investigated systems, compared to the method based on the quench-cooling of the physical mixtures.

### 3.3. Thermal Properties, API Content, and Long-Term Physical Stability Studies of POS-Based Extrudates

A 40D, 12 mm corotating twin-screw extruder, with an air-cooled conveying belt, was used to produce POS-based extrudates containing the API and different types of polymer. The appropriate amount of polymers for HME was chosen based on the rheological data presented in the previous section, i.e., POS + 50% K30, POS + 70% KVA, POS + 50% PXP, and POS + 50% KIR. HME of systems containing K30, KVA, and KIR went smoothly, confirmed by low torque values and engine loads registered during extrusion, i.e., below 3.5 Nm and lower than 50%, respectively. The process outputs for all systems are given in Table 1.

It has to be noted that the HME process of the POS + PXP system was troublesome. During this process, the melt viscosity was frequently changing, which can be connected to the changes in the ratio between API and PXP. At the end of the process, a melt with fluid-like viscosity leaked from the extruder die. This was probably the melted fraction of API, in which a lack of polymer appears—the phase separation. Our suspicion was confirmed by the POS’s content analysis, indicating that the concentration of API in the extrudates with PXP was significantly lower than the employed POS input.

**Figure 7:**
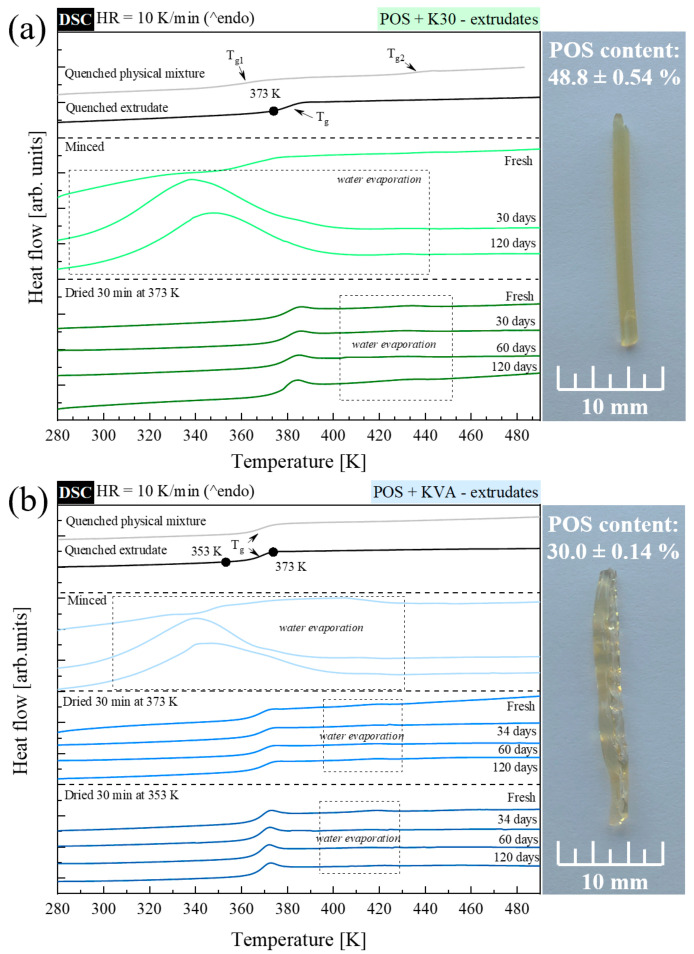
DSC thermograms of (**a**) POS + 50% K30 quenched physical mixture—gray line; quenched extrudate—black line; minced and non-dried extrudate (fresh, and stored for 30 and 120 days)—light green lines; minced and dried for 30 min at 373 K extrudates (fresh, and stored for 30, 60, and 120 days)—dark green lines. DSC thermograms of (**b**) POS + 70% KVA quenched physical mixture—gray line; quenched extrudate—black line; minced and non-dried extrudate (fresh, and stored for 34, and 120 days)—light blue lines; minced and dried for 30 min at 373 K extrudates (fresh, and stored for 34, 60, and 120 days)—intermediate blue lines; and minced and dried for 30 min at 353 K extrudates (fresh, and stored for 34, 60, and 120 days)—dark blue lines. On the right-hand side, POS-based extrudates are shown.

**Figure 8:**
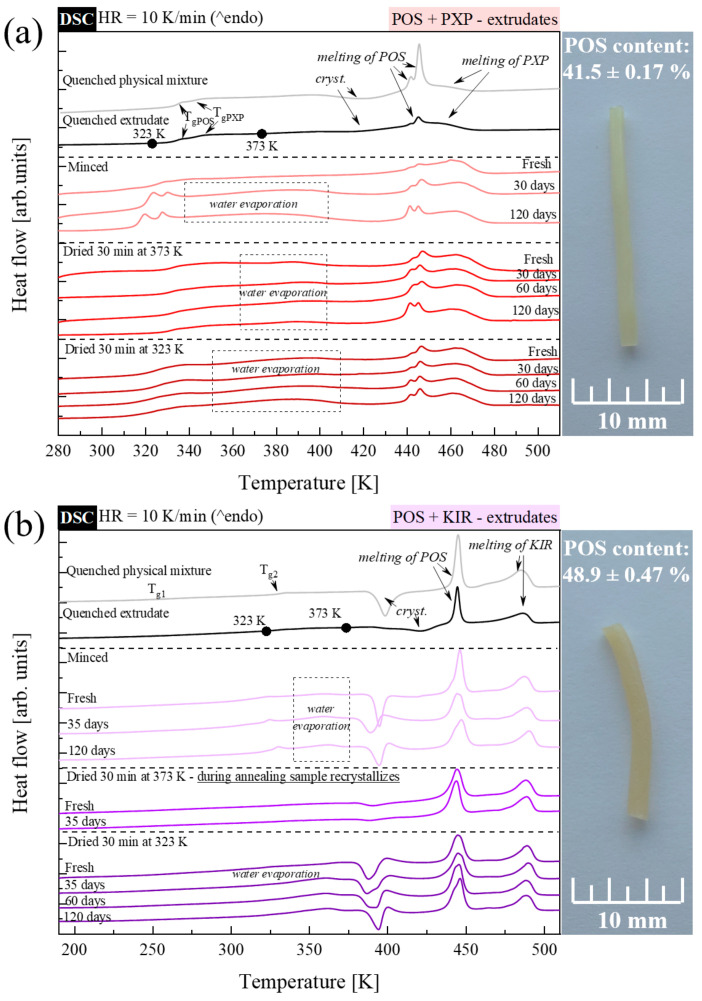
DSC thermograms of (**a**) POS + 50% PXP quenched physical mixture—gray line; quenched extrudate—black line; minced and non-dried extrudate (fresh, and stored for 30 and 120 days)—light red lines; minced and dried for 30 min at 373 K extrudates (fresh, and stored for 30, 60, and 120 days)—intermediate red lines; and minced and dried for 30 min at 323 K extrudates (fresh, and stored for 30, 60, and 120 days)—dark red lines. DSC thermograms of (**b**) POS + 50% KIR quenched physical mixture—gray line; quenched extrudate—black line; minced and non-dried extrudate (fresh, and stored for 5 and 120 days)—light purple lines; minced and dried for 30 min at 373 K extrudates (fresh, and stored for 35 days)—intermediate purple lines; and minced and dried for 30 min at 323 K extrudates (fresh, and stored for 35, 60, and 120 days)—dark purple lines. On the right-hand side, POS-based extrudates are shown.

To investigate the thermal properties of freshly prepared extrudates and those that were stored for up to 120 days, a series of experiments was performed, utilizing standard and temperature-modulated DSC techniques. Extrudates were stored in a glove box at T = 298 K and RH = 25%. Just before all the calorimetric studies, extrudates were minced in the agate mortar. Next, the powdered materials were measured in a few ways. One type of experiment involved heating the tested material, to investigate the sample ‘as obtained’. Such experiments were performed utilizing both the standard DSC technique and TMDSC. The second and third type of experiments included a 30 min annealing step before the standard DSC heating procedure. The annealing aimed to dry the sample. In the second type of experiment, the annealing temperature (T_a_) was equal to 373 K. This temperature, however, is lower than the system’s T_g_ only for compositions containing the K30 polymer—in other cases, during the annealing step, samples were stored at the supercooled liquid region. Thus, to dry the extrudate containing KVA, PXP, and KIR, also at T < T_g_, additional measurements (the third type of experiment) were performed. These experiments included 30 min sample annealing, at T_a_ = 353 K for the system containing KVA, or at T_a_ = 323 K for systems containing PXP or KIR, prior to the main heating step. The obtained DSC thermograms are compared in Figure 7 and Figure 8 for POS + K30 or KVA, and POS + PXP or KIR extrudates, respectively. At the top of each panel, a comparison of the thermograms of the quenched fresh extrudate and quenched physical mixture is presented.

The POS-based extrudates containing the amorphous polymers (50 % of K30 or 70% of KVA) display similar behavior, thus, will be described simultaneously. Fresh, minced samples are characterized by being slightly covered by the water-evaporating endotherm, a glass transition event. During the storage time, the systems take water in, therefore, DSC thermograms measured after 30 or more days show only one broad endothermal peak, associated with the evaporation of the absorbed water. The DSC curves of the quenched, or dried, extrudates, and their reversible signal obtained from TMDSC, display only one clear glass transition event, proving that by employing the HME technique, both polymers in the chosen concentrations form homogeneous compositions. The lack of exotherm following crystallization, and POS’s melting endotherm on each thermogram, indicates that POS, in composition with either K30 or KVA, is physically stable for a minimum of 120 days of storage at T = 298 K and HR = 25%. As the images presented on the right-hand side of Figure 7a,b show, both API + polymer systems form light-yellowish transparent extrudates. The shape of the POS + KVA extrudate is not as perfect as POS + K30, which is associated with too slow water evaporation from this system during the extrusion process.

To the unaided eye, a POS-based extrudate that has been melt-quenched, and should contain 50% semi-crystalline PXP polymer, appears more physically stable than its quenched physical mixture. This is because the DSC thermogram of this sample displays a less pronounced exothermal event associated with the recrystallization process, as well as the melting endotherm reflecting the API melting, when comparing it to the thermal events visible on the DSC thermogram of the quenched physical mixture. In the beginning, this might be confusingly associated with better physical stability, connected with better miscibility. However, considering that the phase separation between POS and PXP exists, and the lower-than-expected content of API in the extrudate was proved, the lower enthalpy of fusion of POS should rather be assigned to the lower API content. Due to the lack of miscibility of POS and PXP, we will not discuss the impact of storage time on the extrudate’s physical stability. Nevertheless, it needs to be stressed that, even after 120 days of storage, the vast majority of the API remains still amorphous in this formulation. Such a conclusion is based on the clearly visible glass transition of POS, in both non-dried and dried long-term-stored extrudates.

Interestingly, besides the highest tendency toward recrystallization noticeable during non-isothermal, calorimetric experiments, the extrudate containing POS + KIR maintained its amorphous form for at least 120 days, when stored at T = 298 K and RH = 25%. The thermograms of extrudates containing POS + semi-crystalline polymer KIR are characterized by the presence of as many as six thermal events. Looking from low to high temperatures, one can distinguish: two glass transition events, a water evaporation endotherm, a crystallization exotherm, and two melting endotherms. The barely seen T_g_, located at 238 K, originates from the small fraction of regions in the polymer displaying a low T_g_. The second glass transition takes the averaged value of the POS’s T_g_ and the second glass transition (i.e., T_g2_) of KIR polymer. The recrystallization process is associated with the devitrification of the API from the system. Since its enthalpy was significantly different for samples annealed at T = 373 K, such experiments were abandoned after 35 days. The visible reduction in the crystallization enthalpy was associated with the recrystallization of POS, that proceeded during the annealing (T = 373 K) step. First, i.e., located at a lower temperature, the melting endotherm reflects the POS melting, while the second endothermal event, which is noticeable at ca. 470 K, originates from the melting of the crystalline fraction of KIR. As can be seen on the images presented on the right-hand side of Figure 8, both POS + PXP and POS + KIR form well-shaped white-yellowish extrudates. Lack of transparency is associated with the employment of semi-crystalline polymers in these systems.

### 3.4. Impact of Polymers on the Aqueous Solubility and Dissolution Rate of POS

The aqueous solubility and dissolution measurements of the POS-based extrudates show the differences between the solubilizing efficacy of the utilized polymers. The solubility of crystalline POS was below the detection limit for the analytical method (LOD = 0.02 µg/mL). The measured solubility values obtained for the physical mixtures and extrudates are presented in Table 2. The highest solubility was obtained for the POS + PXP system. This result, however, cannot be taken as credible, because this extrudate revealed significant discrepancies between the assumed and achieved POS concentrations. In other words, we cannot discuss the solubility of POS + 50% PXP, because the final extrudate is characterized by a different drug content. The averaged sample, i.e., obtained by whole pulverization, contains not 50 but 41.5% of the API. However, it must be stressed that PXP certainly reveals a tendency for significant improvement of POS solubility. This is not the first case when poly(vinyl alcohol) can enhance solubility even more than 150 times [62,63]. A possible mechanism that could explain this, is the formation of polymeric micelles, inside which drug molecules are enclosed [64].

Excluding the POS + PXP system, the best solubility improvement was achieved using K30—an amorphous polymer. It was 7.1 times higher in comparison with the composition containing crystalline POS. Despite the high amount (70%) of the KVA in the POS + KVA system, the solubility, and its improvement, were smaller than those of K30.

**Figure 9:**
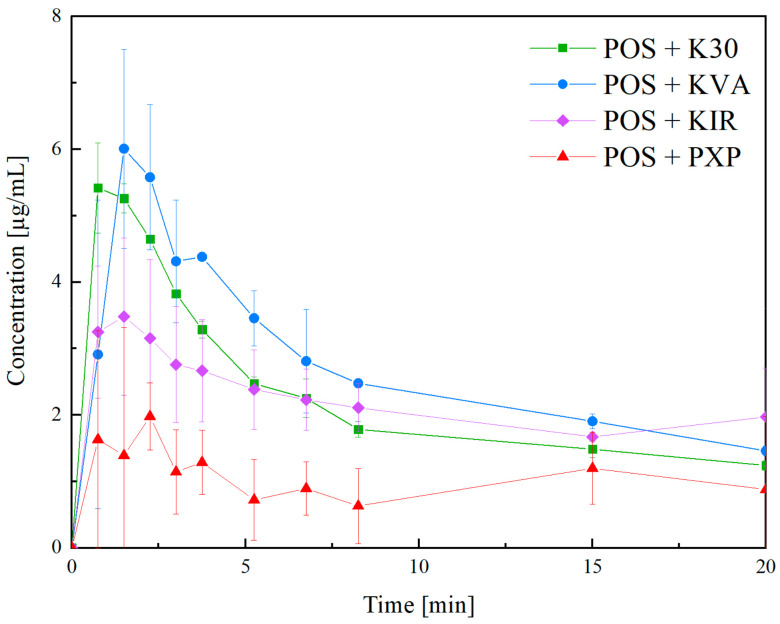
The dissolution profiles of POS + K30, POS + KVA, POS + KIR, and POS + PXP extrudates.

The dissolution studies were performed in water, which creates unfavorable dissolution conditions for POS, but it was a differentiating dissolution medium. The dissolution profiles are presented in Figure 9. The highest temporary concentrations were observed for the POS + KVA and POS + K30 systems. This effect may be attributed to good solubility of the employed amorphous polymers in the dissolution medium. Sun and Lee have shown that the dissolution from matrices made with soluble polymers is characterized by the highest supersaturation effect, while for insoluble materials, diffusion-controlled dissolution is more likely to be observed [64].The supersaturating effect is also visible in the case of the POS + KIR system, as KIR is also water soluble, however, the level of supersaturation is smaller, which may be caused by the slower dissolution of KIR compared to the K30 and KVA polymers. Surprisingly, despite the highest solubility, the lowest POS concentrations were observed for the POS + PXP system. The differences in the dissolution profiles can be attributed to the polymer’s properties, and thus dissolution mechanisms, of the produced amorphous solid dispersions. It can be assumed that, as the PXP can create a gel layer, which acts as the diffusion barrier for API, the carrier-controlled release may be expected, which often results in a slower release, because the API molecules have to diffuse through the gel layer. For solid dispersions with K30, KVA, and KIR, the congruent release mechanism is more likely, which leads to supersaturation followed by recrystallization [65]. The observed decrease in the posaconazole concentrations may be attributed to the recrystallization of the API during the dissolution process. As we have used only a selected particle size fraction (100–200 µm) in our dissolution studies, we can exclude the effect of fine particle dissolution at the first timepoints. Differences in the dissolution profiles were observed in the first 20 min, these were caused by the recrystallization of the amorphous POS; after this time, the amount of dissolved API was comparable for all of the extrudates. As posaconazole has pH-dependent solubility, it has to be highlighted that, together with the development of the dosage form, the dissolution and solubility experiments should also be conducted in media with various pH values, i.e., 1.2, 4.5, and 6.8, and preferably in more biorelevant dissolution media, such as FaSSIF (fasted state simulated intestinal fluid), and FeSSIF (fed state simulated intestinal fluid), as the pH plays an important role in the dissolution rate of posaconazole.

All properties investigated in this paper are summarized in Table 3. The table includes the following criteria: (a) appearance of melting point depression of POS in the physical mixture containing crystalline API, (b) complete miscibility of the API and the employed polymer, reflected by the presence of a single glass transition event in the amorphous form of the physical mixture, (c) lack of recrystallization during the non-isothermal calorimetric studies in any investigated concentration of the POS + polymer melt-quenched system, (d) POS solubility improvement in the physical mixture containing the crystalline API, (e) reaching a drug loading > 30% for the API + polymer system, revealing a melt viscosity suitable for HME, (f) complete miscibility of the API and the employed polymer, that is reflected by the presence of a single glass transition event in the extrudate form, (g) lack of significant modifications in the glass transition event observed after up to 120 days of storage at T = 298 K and RH = 25%, (h) POS solubility improvement in the form of an extrudate, (i) improvement in the POS dissolution rate after the API amorphization, (j) content of POS in extrudate that agrees with the employed API input. The used amorphous polymers (K30 and KVA) are unquestionably superior to the semi-crystalline (PXP and KIR) ones in the formation of efficient POS-based ASDs, according to a 0–1 (does not exist–exists) evaluation of the given criteria. KVA, compared to K30, more easily forms homogeneous systems with POS, and better stabilizes the amorphous form of the investigated API. While K30 polymer, in comparison to KVA, enables the employment of a higher drug content and reveals better solubility, despite using less polymer content. KIR (i.e., the employed representative of semi-crystalline copolymers) is superior among the semi-crystalline polymers. The reason for this is the lack of miscibility of PXP with POS, which was evidenced by the phase separation visible in the thermograms, and the lack of appropriate API content in the extrudates.

## 4. Conclusions

In this paper, the impacts of four different polymers (Kollidon K30 (K30), Kollidon VA64 (KVA), Parteck MXP (PXP), and Kollicoat IR (KIR)) on the physical stability, extrudability, aqueous solubility, and dissolution rate of the amorphous form of posaconazole (POS) were investigated. The aforementioned polymers represent four groups of polymers: (i) K30—amorphous homopolymers, (ii) KVA—amorphous heteropolymers, (iii) PXP—semi-crystalline homopolymers, and (iv) KIR—semi-crystalline heteropolymers. The performed studies prove that amorphous polymers (KVA and K30) are undeniably better excipients than semi-crystalline polymers (KIR and PXP), for formulations containing amorphous POS. These types of polymers guarantee homogeneity and high physical stability when mixed with the POS. The POS-based extrudates obtained with K30 and KVA are transparent, and show between ca. 4 to 7 times better POS aqueous solubility than their physical mixtures (i.e., the counterparts containing the crystalline API). The main differences in the efficiencies of the tested amorphous polymers in creating POS-based ASDs suitable for HME, result from the ease of creating a homogeneous system and drug loading. Employing K30, a higher amount of drug can be used to obtain a melt viscosity suitable for HME, compared to utilizing KVA, while the KVA guarantees better homogeneity. Moreover, POS K30 extrudate is characterized by uniform shape and good mechanical strength, which leads to the conclusion that it may be utilized as a filament in fused deposition modeling 3D printing. The main disadvantages of the utilized semi-crystalline polymers are, the lack of POS melting point depression in their systems, and the lack of complete homogeneity or even miscibility, as in the case of the POS + PXP system. Despite the worst improvement in solubility, the KIR—the representative heteropolymer—was ranked as the third excipient for the formulation of POS-based ASDs. This is because POS extruded with KIR is characterized by an appropriate content, and reveals completely amorphous form, for at least 120 days of storage at standard storage conditions. PXP cannot fully protect POS from recrystallization and form a miscible system with it. Nevertheless, in the future, it would be worth exploring the mechanism responsible for this semi-crystalline homopolymer improving POS’s aqueous solubility.

## Figures and Tables

**Table 1:** HME process outputs for POS + K30, POS + KVA, POS + PXP, and POS + KIR systems.

Binary System	API Concentration (%) ± SD	Extrusion Temp. (K)	Max Pressure (MPa)	Max. Torque (Nm)	Max. Engine Load (%)
POS + 50% K30	48.8 ± 0.54	443	0.14	3.2	39.1
POS + 70% KVA	30.0 ± 0.14	443	0.23	2.5	30.9
POS + 50% KIR	48.9 ± 0.47	443	1.88	3.4	42.4
POS + 50% PXP	41.5 ± 0.17	463	0.08	3.0	37.4

**Table 2:** The measured aqueous solubility values obtained for the physical mixtures and extrudates.

Binary System	POS Solubility (µg/mL)		Improvement Factor
	Physical Mixture	Extrudate	
POS + 50% K30	0.05	0.36	7.1
POS + 70% KVA	0.08	0.30	3.7
POS + 50% KIR	0.02	0.05	2.8
POS + 50% PXP	0.03	4.58	163.4

**Table 3:** Comparison of all properties investigated in the paper: (a) appearance of melting point depression, (b) complete miscibility of POS and polymer, (c) lack of recrystallization during the non-isothermal calorimetric studies in the POS + polymer melt-quenched system, (d) POS solubility improvement in the physical mixture containing crystalline API, (e) reaching a drug loading > 30% for the system, revealing melt viscosity suitable for HME, (f) complete miscibility of the API and polymer in extrudate, (g) lack of significant modifications in T_g_ observed after up to 120 days of storage at T = 298 K and RH = 25%, (h) improvement of POS solubility being in the form of extrudate, (i) improvement in the POS dissolution rate after the API amorphization, (j) content of POS in extrudate that agrees with the employed API input.

Polymer	(a)	(b)	(c)	(d)	(e)	(f)	(g)	(h)	(i)	(j)
K30	1	0	1	1	1	1	1	1	1	1
KVA	1	1	1	1	0	1	1	1	1	1
PXP	0	0	0	0	1	0	0	1	0	0
KIR	0	0	0	0	1	0	1	1	0	1

## Data Availability

Data is contained within the article or Supplementary Material.

## Supplementary Materials

The following supporting information can be downloaded at: https://www.mdpi.com/article/10.3390/pharmaceutics15030799/s1, Figure S1: TGA of neat POS; Figure S2: TGA of (a) neat K30, and POS + 50% K30; (b) neat KVA and POS + 70% KVA; (c) neat PXP and POS + 50% PXP; (d) neat KIR and POS + 50% KIR; Figure S3: Zoomed area of Figure 3a, showing separation of Tgs and POS’s recrystallization from the systems containing POS + K30; Figure S4: Zoomed area of Figure 3b, showing recrystallization of POS from the system containing POS + 10% KVA; Figure S5: Zoomed area of Figure 3c, showing separation of POS and PXP glass transitions in the systems of POS + PXP; Figure S6: Zoomed area of Figure 3d, showing appearance of two glass transition events in the neat KIR polymer, as well as the influence of POS on their values; Figure S7: DSC thermograms obtained on cooling, with a rate of 20 K/min, of melted physical mixtures containing POS and, from 10–70wt.%, of (a) Kollidon 30 (K30), (b) Kollidon VA64 (KVA), (c) Parteck MXP (PXP), and (d) Kollicoat IR (KIR). In each panel, the higher thermogram represents a DSC trace of neat POS, while the lowest thermogram originates from neat polymer.

## Abbreviations

API	Active pharmaceutical ingredients
ASD	Amorphous solid dispersions
BCS	Biopharmaceutical classification system
DSC	Differential scanning calorimetry
FaSSIF	Fasted state simulated intestinal fluid
FeSSIF	Fed state simulated intestinal fluid
HME	Hot melt extrusion
HPLC	High-performance liquid chromatography
HPMC	(Hydroxypropyl)methyl cellulose
HPMCAS	Hypromellose acetate succinate
K30	Kollidon 30
KIR	Kollicoat IR
KVA	Kollidon VA64
NCE	New chemical entity
PEG	Polyethylene glycol
POS	Posaconazole
PSSA	Poly(styrene sulfonic acid)
PVA	Poly(vinyl alcohol)
PVP	Poly(vinylpyrrolidone)
PVPVA	Poly(vinylpyrrolidone-vinyl acetate)
PXP	Parteck MXP
SD	Standard deviations
SOP	Soluplus
TMDSC	Temperature modulated differential scanning calorimetry
