# Supplementary material for: Hot Melt Extruded Posaconazole-Based Amorphous Solid Dispersions—The Effect of Different Types of Polymers
Source: Pharmaceutics. 2023 Feb 28;15(3):799. doi: 10.3390/pharmaceutics15030799 (PMC10056184; doi:10.3390/pharmaceutics15030799)
Supplement: Supplementary file 1 [file pharmaceutics-15-00799-s001.zip › pharmaceutics-2227922-supplementary.pdf]

Supplementary Materials

Hot melt extruded posaconazole-based amorphous solid dispersions – the effect of different types of polymers

Daniel Kramarczyk [1,*], Justyna Knapik-Kowalczuk [1,*], Mateusz Kurek [2], Witold Jamróz [2], Renata Jachowicz [2] and Marian Paluch [1]

[1] Faculty of Science and Technology, Institute of Physics, University of Silesia in Katowice, SMCEBI, 75 Pułku Piechoty 1a, 41-500 Chorzów, Poland;
[2] Faculty of Pharmacy, Department of Pharmaceutical Technology and Biopharmaceutics, Jagiellonian University, Medyczna 9, 30-688 Kraków, Poland;
***** Correspondence: daniel.kramarczyk@smcebi.edu.pl (DK); justyna.knapik-kowalczuk@us.edu.pl (JKK), Tel.: 0048 32 3497629;

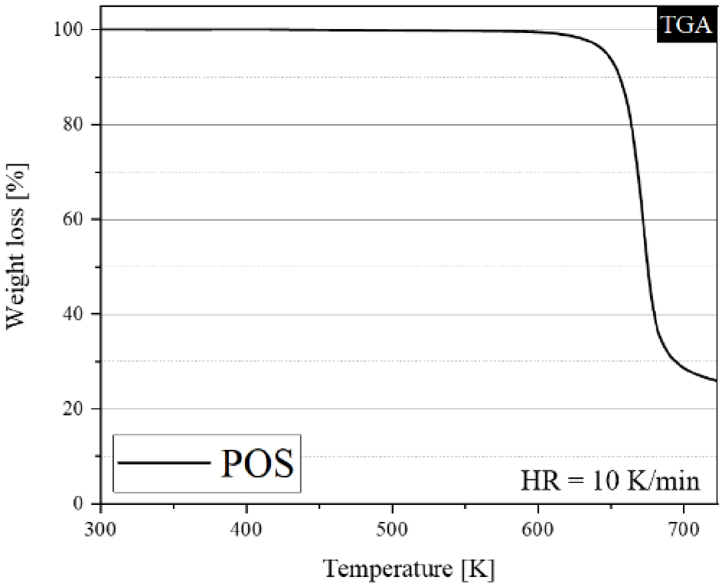

Figure S1. TGA of neat POS

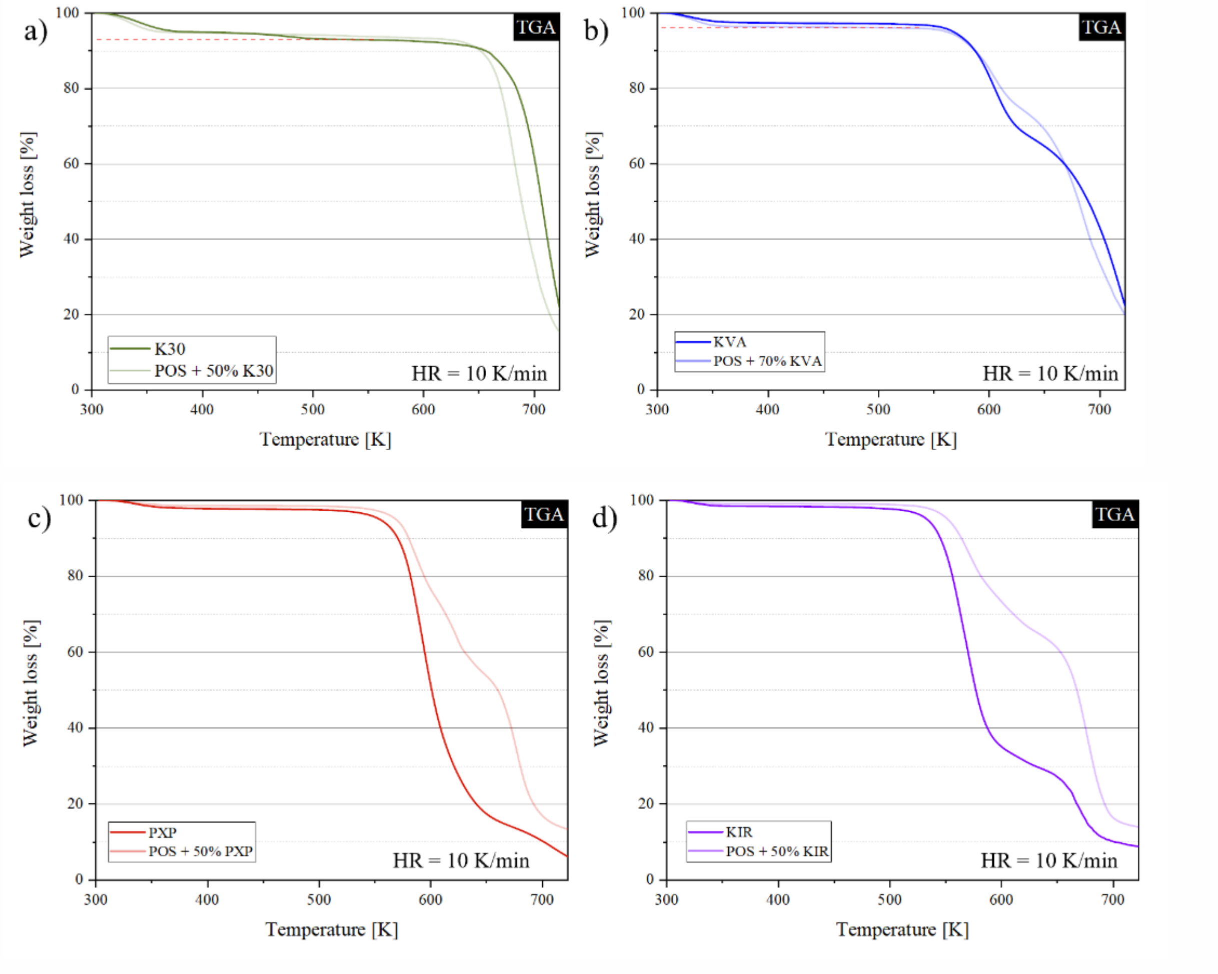

Figure S2. TGA of (a) neat K30, and POS + 50% K30; (b) neat KVA and POS + 70% KVA; (c) neat PXP and POS + 50% PXP; d) neat KIR and POS + 50% KIR

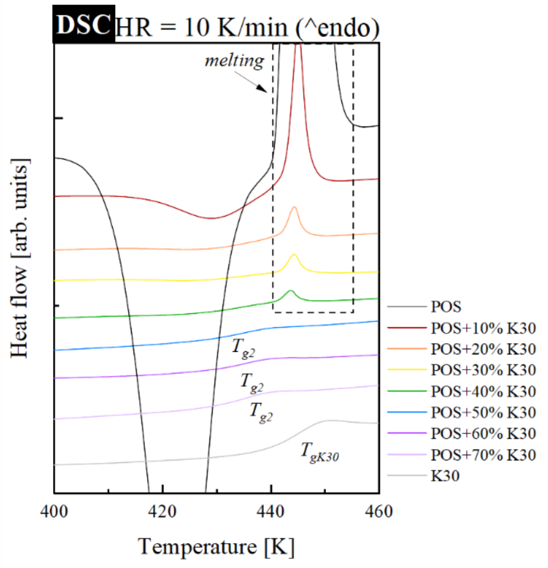

Figure S3. Zoomed area of Figure 3a showing separation of Tgs and POS's recrystallization from the systems containing POS + K30

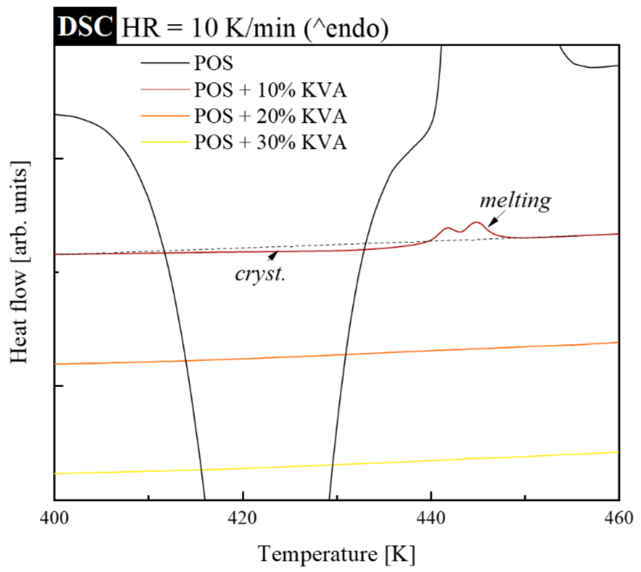

Figure S4. Zoomed area of Figure 3b showing recrystallization of POS from the system containing POS + 10% KVA

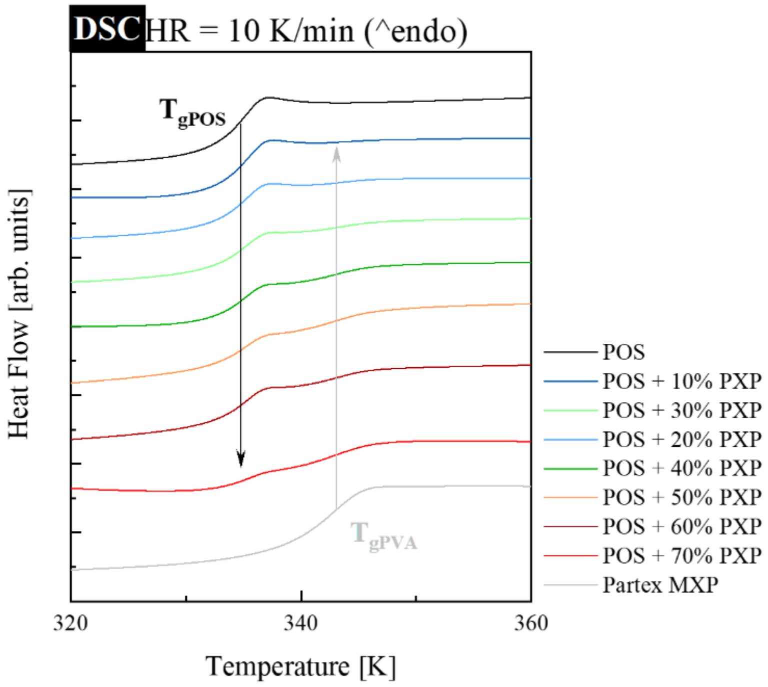

Figure S5. Zoomed area of Figure 3c showing separation of POS and PXP glass transitions in the systems of POS + PXP

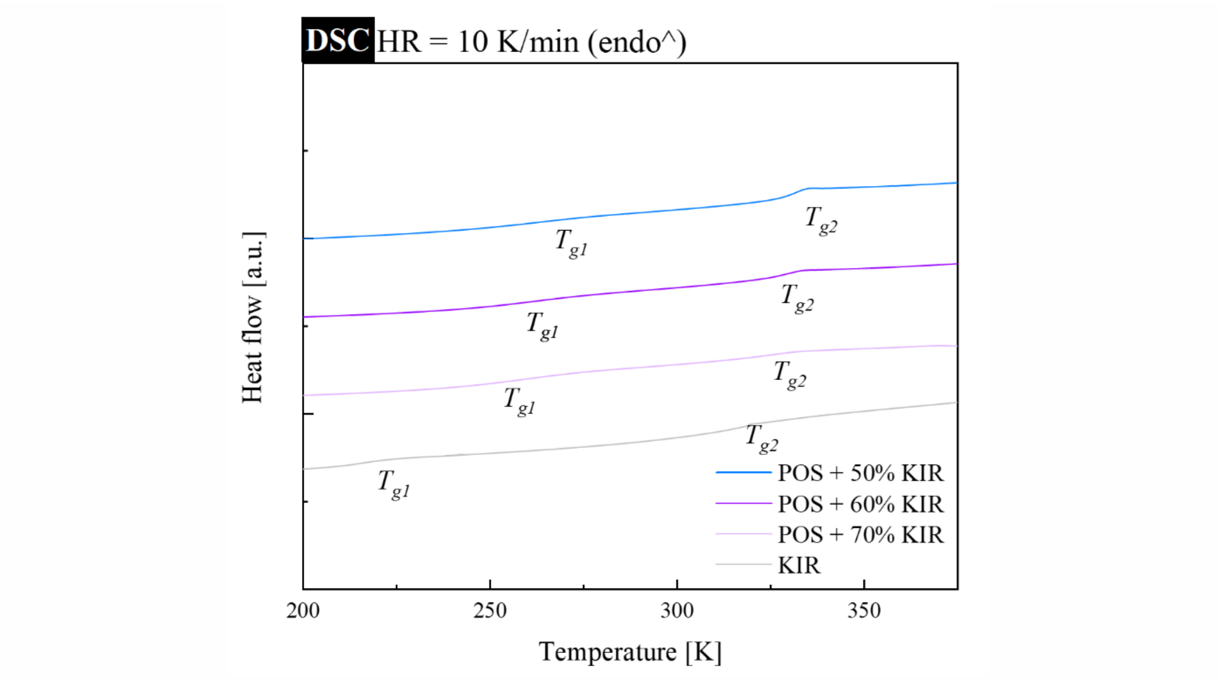

Figure S6. Zoomed area of Figure 3d showing appearance of two glass transition events in the neat KIR polymer, as well as the influence of POS on their values

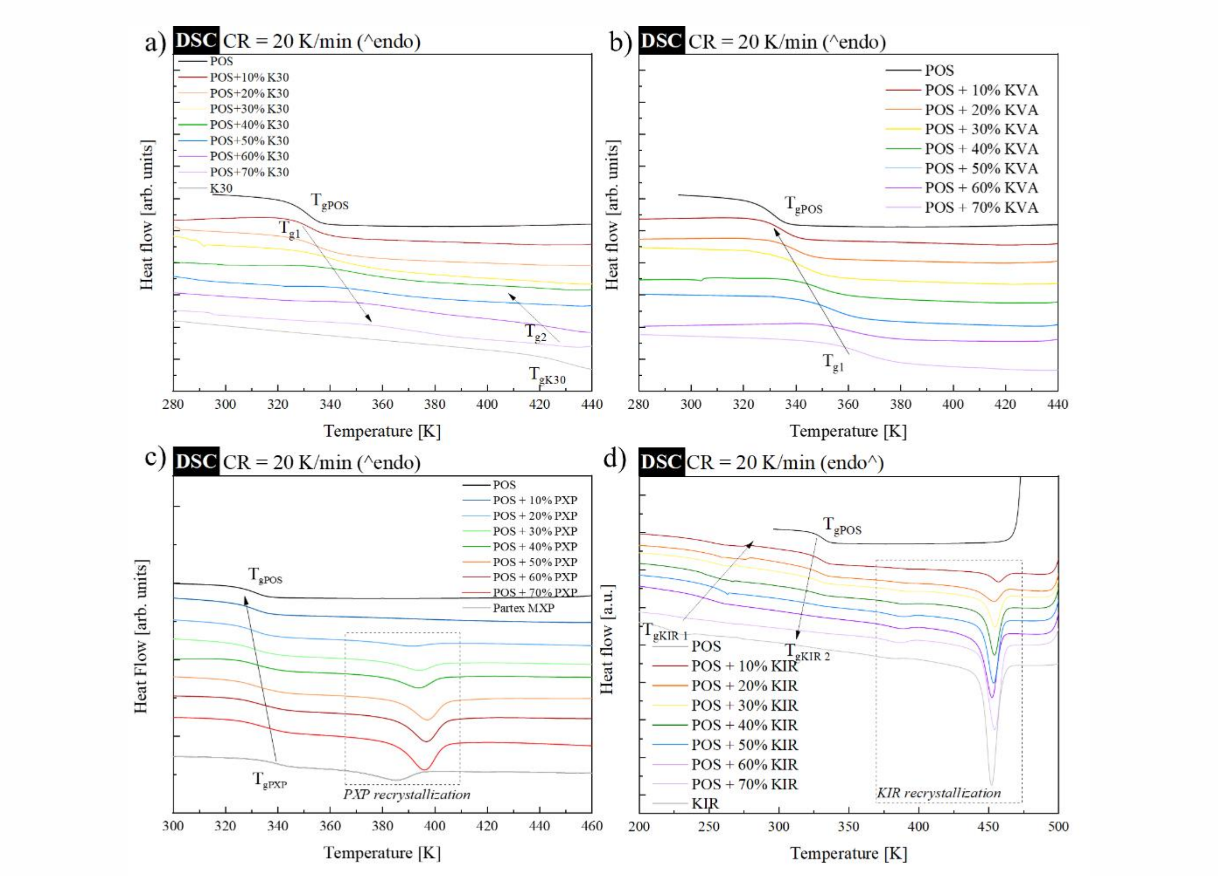

Figure S7. DSC thermograms obtained on cooling with a rate of 20 K/min of melted physical mixtures containing POS and, from 10 – 70wt. %, of (a) Kollidon 30 (K30), (b) Kollidon VA64 (KVA), (c) Parteck

MXP (PXP), and (d) Kollicoat IR (KIR). In each panel, the higher thermogram represents a DSC trace of neat POS, while the lowest thermogram originates from neat polymer.
